# Factors influencing productivity of eastern wild turkeys in northeastern South Dakota

**DOI:** 10.1002/ece3.6583

**Published:** 2020-07-23

**Authors:** Reina M. Tyl, Christopher T. Rota, Chadwick P. Lehman

**Affiliations:** ^1^ Division of Forestry and Natural Resources West Virginia University Morgantown WV USA; ^2^ South Dakota Game, Fish and Parks Custer SD USA; ^3^ Missouri Department of Conservation Central Regional Office and Conservation Research Center Columbia MO USA

**Keywords:** clutch size, hatchability, nest survival, nesting rate, poult survival, renesting rate

## Abstract

Population growth is highly sensitive to changes in reproductive rates for many avian species. Understanding how reproductive rates are related to environmental conditions can give managers insight into factors contributing to population change. Harvest trends of eastern wild turkey in northeastern South Dakota suggest a decline in abundance. We investigated factors influencing reproductive success of this important game bird to identify potential factors contributing to the decline. We monitored nesting rate, nest survival, renesting rate, clutch size, hatchability, and poult survival of 116 eastern wild turkey hens using VHF radio transmitters during the springs and summers of 2017 and 2018. Heavier hens were more likely to attempt to nest than lighter hens, and adult hens were more likely to renest than yearling hens. Nest survival probability was lowest in agricultural fields relative to all other cover types and positively related to horizontal visual obstruction and distance to the nearest road. Daily nest survival probability demonstrated an interaction between temperature and precipitation, such that nest survival probability was lower on warm, wet days, but lowest on dry days. Egg predation was the leading cause of nest failure, followed by haying of the nest bowl and death of the incubating hen. Poults reared by adult hens had a greater probability of survival than poults reared by yearling hens. Our estimate of survival probability of poults raised by yearling hens was low relative to other studies, which may be contributing to the apparent regional population decline. However, there is little managers can do to influence poult survival in yearling hens. Alternatively, we found nest survival probability was lowest for nests initiated in agricultural fields. Wildlife‐friendly harvesting practices such as delayed haying or installation of flushing bars could help increase productivity of eastern wild turkey in northeastern South Dakota.

## Graphical abstract

Eastern wild turkey (Meleagris gallopavo silvestris ) hens and poults move through a pasture in Grant County, South Dakota, USA. Photograph taken by Jenna Wolf and used with permission.

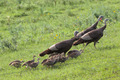

## 1 INTRODUCTION

Population dynamics in closed systems are governed by survival and reproduction (Caswell, 2001). For many avian species, population growth is highly sensitive to changes in reproductive rates (Sæther & Bakke, 2000). Many factors contribute to variation in reproductive rates, and knowledge of how reproductive rates are related to environmental variation can give insight into drivers of population dynamics through time. This can guide management activities for species of conservation concern by identifying environmental variables that are associated with reproductive rates and how incremental changes in these variables are likely to influence reproduction and ultimately population growth (Mills, 2007).

Eastern wild turkey (*Meleagris gallopavo silvestris*; hereafter turkey) are an important game species across North America. This species tends to be relatively short‐lived with high reproductive output (McRoberts, Wallace, & Eaton, 2014), making population growth rates highly sensitive to reproductive rates (Sæther & Bakke, 2000). Studies in New York (Roberts, Coffey, & Porter, 1995) and Wisconsin (Pollentier, Hull, & Lutz, 2014; Rolley, Kubisiak, Paisley, & Wright, 1998) have demonstrated population growth can be highly sensitive to changes in reproductive rates. Understanding factors influencing reproduction in turkey is therefore necessary for effective management of this important game species.

Both intrinsic (e.g., body condition, age) and extrinsic environmental variables influence reproductive rates for turkey. Hen body condition is an important determinant of reproductive success. For example, hen weight can be positively associated with nesting probability and nest success (Porter, Nelson, & Mattson, 1983; Vander Haegen, Dodge, & Sayre, 1988). Similarly, adult hens are more likely to nest and renest than yearling hens (Lehman, Flake, Leif, & Shields, 2001; Paisley, Wright, Kubisiak, & Rolley, 1998; Pollentier, Lutz, & Hull, 2014; Porter et al., 1983; Shields & Flake, 2006; Vander Haegen et al., 1988). Adult hens may also enhance survival probability of their poults relative to yearling hens (Porter et al., 1983). Extrinsic environmental variables can also influence turkey reproductive rates. For example, precipitation can be negatively associated with daily nest survival and survival of poults <2 weeks old (Healy, 1992; Healy & Nenno, 1985; Lehman, Flake, Rumble, & Thompson, 2008; Roberts & Porter, 1998a; Vangilder & Kurzejeski, 1995). In northern populations, cold weather during the brood‐rearing season can be detrimental to poult survival and overall reproductive success (Healy, 1992; Healy & Nenno, 1985).

Although intrinsic and extrinsic environmental variables can be important determinants of turkey reproductive success, managers have little or no ability to influence these variables. In contrast, managers often have some ability to manipulate habitat‐related environmental variables. One habitat‐related environmental variable that can be important to turkey reproductive success is cover type. For example, Clawson and Rotella (1998) found that artificial nests located in Conservation Reserve Program (CRP) fields had greater success relative to nests located in non‐CRP cover types. In contrast, hens nesting in agricultural cover types such as alfalfa fields may be subject to increase risk of nest failure and hen mortality (Paisley et al., 1998; Shields & Flake, 2006; Vangilder & Kurzejeski, 1995). South Dakota has seen large‐scale landscape changes as grasslands have been converted into row crop or other agricultural cover types, with the greatest losses having occurred in the northeastern region (16.9% between 2006 and 2012) (Reitsma et al., 2014). This loss of grassland cover types parallels declines in the amount of land enrolled in CRP (Hellerstein, 2017), which has been particularly steep in northeastern South Dakota (USDA, 2016). Microhabitat conditions can also be strongly associated with reproductive rates. For example, increased visual obstruction can be positively associated with nest success (Badyaev, 1995; Lutz & Crawford, 1987), likely because of reduced detectability by predators.

For this study, we evaluated factors influencing reproductive success of turkeys in northeastern South Dakota. Harvest of turkeys in northeastern South Dakota during the spring prairie turkey season declined more than 50% between 2010 and 2016 (Huxoll, 2016), prompting managers to study potential causes of this apparent decline. Since productivity has a strong influence on population growth of this species (Pollentier, Hull, et al., 2014; Roberts et al., 1995; Rolley et al., 1998), understanding the factors that influence reproduction in this population is necessary to identify and potentially reverse the causes of this apparent decline. The objectives of this study are to (a) obtain baseline estimates of nesting rate, nest survival, renesting rate, clutch size, and hatchability; (b) obtain estimates of poult survival over the 28‐day posthatch interval; and (c) determine the effects of intrinsic and environmental variables on nest and poult survival for turkey hens in northeastern South Dakota. The results of this study will improve understanding of the factors influencing turkey productivity in open, agriculturally dominated landscapes and inform management of turkeys in northeastern South Dakota.

## 2 MATERIALS AND METHODS

### 2.1 Study area

The study was conducted in Codington, Deuel, Grant, and Roberts counties in northeastern South Dakota. The study area was split between the Minnesota River‐Red River Lowland in the eastern half of the study area and Coteau des Prairies physiographic region in the western half (Flint, 1955; Johnson, Higgins, & Hubbard, 1995). The Coteau begins in the northwest and extends in a southeasterly direction across the study area (Miller, Kempf, & Koopman, 1979). On top of the Coteau, the relief is gently undulating to hilly, while down in the Lowlands the land is nearly level (Flint, 1955; Miller et al., 1979). Elevations ranged from over 600 m above mean sea level on top of the Coteau to about 300 m above sea level in the Lowland (Miller et al., 1979). Most of the study area consisted of privately owned lands with some state‐owned (e.g., Game Production Areas) and federally owned (e.g., Waterfowl Production Areas) lands scattered throughout.

**FIGURE 1:**
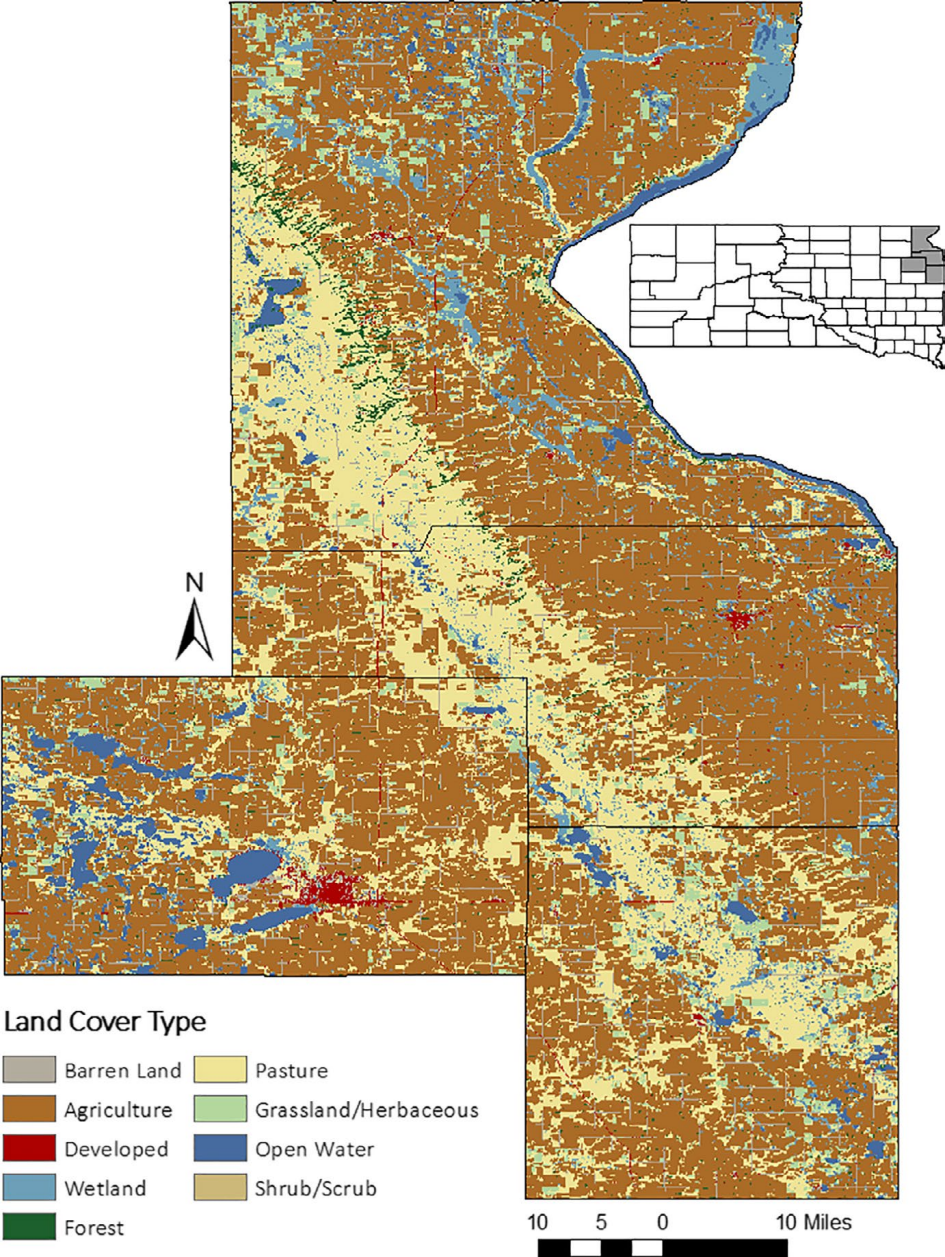
Map of land cover types (adapted from the National Land Cover Database 2016 land cover raster layer; Yang et al. 2018) in Codington, Deuel, Grant, and Roberts Counties in northeastern South Dakota, USA

Agriculture dominates land use in the study area, with most land being used for either cropland, rangeland, or to grow alfalfa or hay for livestock feed (Miller et al., 1979) (Figure 1). Most of the grain farming (i.e., corn and soybeans) occurs in the Lowland (Miller et al., 1979). The highlands of the Coteau support native tallgrass prairie which is used primarily for rangeland; however, scattered fields of hay and alfalfa are in the highlands as well (Miller et al., 1979). Common grasses include warm‐season grasses such as big bluestem (*Andropogon gerardii*), little bluestem (*Schizachyrium scoparium*), Indiangrass (*Sorghastrum nutans*), switchgrass (*Panicum virgatum*), and sideoats grama (*Bouteloua curtipendula*) (Johnson & Larson, 2007). Common cool‐season grasses include smooth brome (*Bromus inermis*), Kentucky bluegrass (*Poa pratensis*), western wheatgrass (*Pascopyrum smithii*), and green needlegrass (*Stipa viridula*) (Johnson & Larson, 2007). Numerous forbs and patches of western snowberry (*Symphoricarpos occidentalis*) are scattered throughout the pasture lands (Johnson & Larson, 2007). Forested areas along the east‐facing breaks where the Coteau descends into the Lowlands are dominated by bur oak (*Quercus macrocarpa*) on the drier slopes (Leatherberry, Piva, & Josten, 2000). More mesic areas are dominated by elm‐ash (*Fraxinus* spp.; *Ulmus* spp.) forests (Leatherberry et al., 2000) that are intermixed with trembling aspen (*Populus tremuloides*), box elder (*Acer negundo*), eastern cottonwood (*Populus deltoides*), and sugar maple (*Acer saccharum*) (Knupp Moore & Flake, 1994).

**FIGURE 2:**
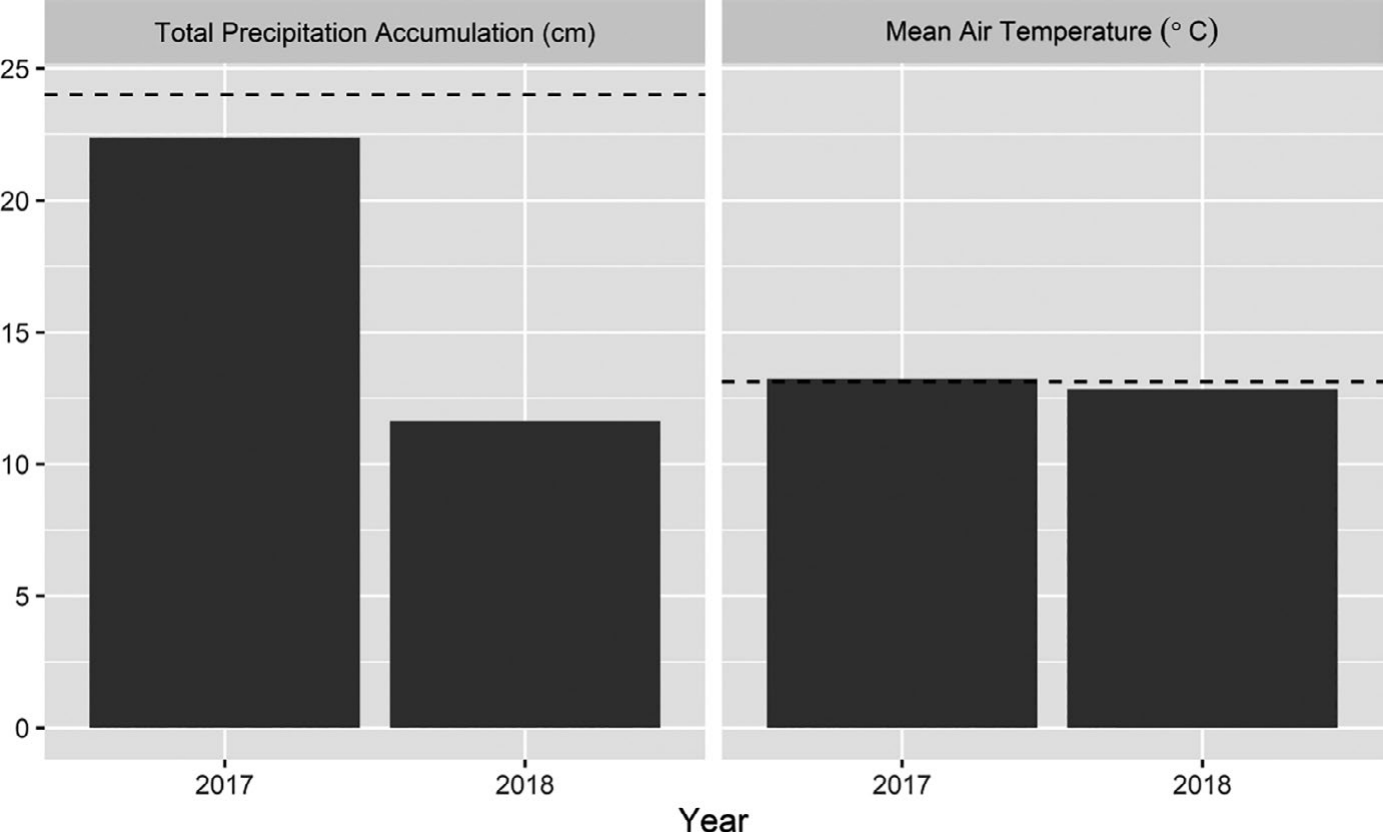
Total precipitation accumulation (i.e., rainfall) (cm) and mean air temperature (°C) during the springs (1 April to 30 June) of 2017 and 2018 in northeastern South Dakota, USA. The 30‐year average (1989–2018) for total precipitation accumulation (24.0‐cm) and mean air temperature (13.1°C) during spring in Milbank, South Dakota, USA, are indicated by the horizontal dashed lines (Menne et al., 2012)

Northeastern South Dakota is in a humid continental climate region, with mean annual precipitation of 57 cm and mean annual temperature of 6.5°C across the study area (Menne et al., 2012). Early spring snowfall is possible, with about one‐quarter (26%) of the total annual snowfall occurring from March through May (Menne et al., 2012). About 60% of the total annual precipitation occurs during the nesting and brood‐rearing seasons (April through August; Menne et al., 2012). Northeastern South Dakota received below average precipitation (i.e., rainfall) and approximately average temperatures during spring seasons (1 April–30 June) over the course of this study (Figure 2). Spring and summer temperatures can be highly variable, with average minimum temperatures near 0°C in early spring to average maximum temperatures near 28°C during summer; however, normal mean temperatures for the spring and summer months range from 13 to 19°C (Menne et al., 2012).

### 2.2 Capture and radio telemetry

We monitored reproduction of turkeys by fitting female turkeys with radio transmitters. We captured turkeys by first locating flocks of turkeys during the winter (1 January–31 March) and then baiting turkeys into capture sites. We captured turkeys using rocket nets (Thompson & Delong, 1967; Wunz, 1984). Following capture, we aged female turkeys as adult or yearling based on the presence or absence of barring on the 9th and 10th primary feathers (Williams, 1961) and weighed each bird. We secured 80‐g very high‐frequency (VHF) radio telemetry transmitters (Advanced Telemetry Systems) using a shock cord harness and backpack mount. Transmitters were <3% of the hens' body weight to reduce the risk of the transmitter interfering with survival and reproduction (Fair, Paul, & Jones, 2010). Transmitters were equipped with an activity signal, a nonmoving (loafing) signal that is activated instantaneously whenever the hen is not in motion, and mortality signal set to activate after 8 hr of inactivity. We monitored turkeys 6 days per week during the spring and summer (1 April–31 July) by locating each transmitter signal and listening to the nature of the signal; however, turkeys that were incubating nests were monitored daily. A moving signal indicated the hen was alive but not incubating a nest, a nonmoving signal indicated the hen was alive and incubating a nest, and a mortality signal indicated a hen was no longer alive. All handling, marking, and monitoring procedures were approved by the West Virginia University Institutional Animal Care and Use Committee (Permit No. 1606003205; South Dakota State Permit 37).

### 2.3 Nest marking and monitoring

We monitored nesting activity of hens daily from 1 April to 6 August, 2017–2018. We first determined onset of incubation by listening for nonmoving signals from VHF transmitters. Once a nonmoving signal was obtained, we located nesting hens via homing and marked the nest. We marked the nest area with ~4 flags at distances of 20–40 m from the nest bowl depending on cover height and density of vegetation while attempting to minimize disturbance. If a nonmoving signal was observed on a subsequent day, we assumed that the hen was still tending the nest. If a moving signal was observed, we visually inspected the nest bowl to determine whether the hen was temporarily away (i.e., eggs and nest bowl still active and not disturbed), or whether it was lost due to predation (i.e., smashed or removed eggs). If a mortality signal was observed, we assumed that the hen died while tending the nest and we located the transmitter and assessed the cause of death. We classified nests as successful by the presence of hatched eggshells, or as failed if nest contents were depredated, destroyed, or abandoned (Lehman et al., 2001). If the nest was successful, we determined the number of eggs that hatched from the total clutch size by counting eggshell fragments and membranes (Lehman et al., 2001). We counted the number of eggs in failed nests to determine clutch size if the eggs were relatively intact and undisturbed (Lehman et al., 2001). If the clutch size of failed nests could not be accurately determined, we did not include that nest in the analysis of clutch size.

### 2.4 Poult monitoring

We determined the initial number of poults that hatched from each successful nest based on egg shell and membrane remains (Lehman et al., 2001). The number of poults in each brood was counted at 1, 2, and 4 weeks posthatch by observing broods feeding in open areas (Lehman, Flake, et al., 2008); however, if dense vegetation interfered with observations, broods were flushed to count poults. Broods often formed crèches (multiple hens with a group of commingled poults) after poults were 2 weeks old, and crèches were common when broods were 4 weeks old, making it difficult to differentiate individual broods during the day. If we could not determine the number of poults in a brood during the day due to the formation of a crèche, we performed another poult count for that brood again at night. During night brood counts, we observed the brood while in the roost with the hen, being careful to not flush the group from the roost, to obtain an accurate count of poults (Lehman, Flake, et al., 2008).

### 2.5 Environmental and spatial covariate estimation

We sought to determine how nest‐site characteristics influenced nest success. Therefore, we quantified nest‐site characteristics on the hatch date for successful nests and on the projected hatch date for failed nests (Gibson, Blomberg, & Sedinger, 2016; McConnell, Monroe, Burger, & Martin, 2017; Smith et al., 2018). We measured understory visual obstruction readings (VOR) of vegetation by placing a Robel pole with 2.54 cm increments in the nest bowl and at 1 m from the nest in the four cardinal directions (Benkobi, Uresk, Schenbeck, & King, 2000; Robel, Briggs, Dayton, & Hulbert, 1970). We recorded the lowest visible increment on the pole from a distance of 4 m while kneeling to a height of 1 m (Lehman, Rumble, Flake, & Thompson, 2008; Robel et al., 1970). We measured VOR from the four cardinal directions at the nest bowl; however, at the peripheral 1 m from the nest measurements, we estimated VOR from only three cardinal directions, ignoring the 4th direction back across the nest bowl so as not to duplicate visual obstruction readings across the nest bowl (Lehman, Rumble, et al., 2008). We measured the height (in centimeters) of living vegetation at the nest bowl and at 1 m from the nest in the 4 cardinal directions (Lehman, Rumble, et al., 2008). We estimated total cover of grass, forbs, shrubs, and other cover using a 0.1‐m^2^ quadrat at the nest bowl, and at 5, 1‐m intervals in the cardinal directions (Daubenmire, 1959). We qualitatively categorized the dominant land cover type within the area surrounding the nest bowl as either grassland, pasture, agriculture, or forest. We classified the land cover as pasture if grazing was currently occurring or had occurred that year. Alfalfa hayfields and row crop fields were classified as agriculture. CRP grasslands, old fields, and other land cover where the dominant vegetation was grass and forbs and where grazing did not occur were classified as grasslands. If a nest was located within a road ditch, we classified the land cover according to the dominant land use adjacent to the road ditch (e.g., a nest placed in a road ditch next to a corn field would be classified as agriculture). We used ArcMap version 10.6.1 (Environmental Systems Research Institute) to calculate the distance from each nest to the nearest road (i.e., interstate, federal highway, state highway, local paved road, local unpaved road), obtained from the South Dakota Department of Transportation (SDDOT, 2017).

We placed 10 precipitation and temperature monitoring stations throughout the study area before the onset of nesting and retrieved the monitoring stations after all broods were >4 weeks old. Monitoring stations consisted of a rain gauge and a HOBO Pendant Temperature Data Logger (Onset Computer Corporation) that recorded 4 or 6 temperature readings at evenly spaced intervals each day. Monitoring stations were placed systematically throughout the study area to cover the extent of all radio‐marked hen locations. Rain gauges were checked after every precipitation event, and we calculated daily precipitation accumulation (mm) (hereafter precipitation) and daily mean temperature (°C) (hereafter temperature) for each monitoring station for each day of the study. We assigned precipitation and temperature covariates to each individual nest and each individual brood (for nest and poult survival analyses, respectively) by assuming precipitation and temperature conditions at the location of each nest and at the location of each brood was equal to the precipitation and temperature conditions observed at the closest monitoring station.

### 2.6 Modeling reproductive parameters

#### 2.6.1 Nesting rate

We modeled nesting rate as the probability an individual hen that was alive on 1 April would attempt to nest that year using Bayesian logistic regression. We modeled nesting rate as a function of the age‐class of each hen (adult or yearling), year of the study (2017 or 2018), and weight of each hen (kg). We used informative prior distributions for the intercept (log odds a juvenile initiates a nest when all other coefficients fixed at 0) and the slope coefficient describing the difference in log odds of nesting (i.e., log odds ratio [LOR]) between adult and juveniles. Drawing upon the studies in Table 2, we used a Gaussian (mean = 0.9; *SD* = 0.2) prior distribution for the intercept coefficient and a Gaussian (mean = 1.6; *SD* = 0.8) prior distribution for the adult LOR coefficient. Details on how we derived informative prior distributions are in Appendix 2. We assumed logistic (location = 0; scale = 1) prior distributions for all other slope coefficients.

#### 2.6.2 Nest survival

We assumed survival of nest *i* during day *t* was a Bernoulli random variable:

$$y_{\mathrm{it}}∼\text{Bernoulli}\left(y_{i\left(t‐1\right)}p_{\mathrm{it}}\right)$$

where *y_it_ = *1 if nest *i* survived day *t*, *y_it_ = *0 if nest *i* failed during day *t*, and *p_it_* represents daily survival probability (Royle & Dorazio, 2008). We further assumed a logit‐linear model for daily survival probability which we model as a function of age‐class of the nesting hen (adult or yearling), precipitation, temperature, land cover type (agriculture, forest, grassland, or pasture), mean VOR, mean total cover, and distance to the nearest road (m). We included an interactive effect of precipitation on temperature, since the effect of precipitation may vary depending on the temperature. Fifteen failed nests were missing VOR and total cover observations because the vegetation surrounding the nest was removed via haying before it could be measured. Rather than discard those nests, we imputed missing predictor variables, accounting for uncertainty in unmeasured variables (Gelman et al., 2013).

We accounted for repeated observations on individual nests by fitting a random coefficients model (Gelman & Hill, 2007). We assumed each coefficient *β_ji_* was a Gaussian random variable:

$$\beta_{\mathrm{ji}}∼\text{Gaussian}\left(\mu_{j},\tau_{j}\right)$$

where *µ_j_* and *τ_j_* represent the population‐level mean and precision, respectively, of slope coefficient *j*. We used informative prior distributions for the intercept and adult LOR population‐level mean parameters. Drawing upon the studies in Table 2, we used a Gaussian (mean = 3.2; *SD* = 0.3) prior distribution for the intercept population‐level mean parameter and a Gaussian (mean = 0.3; *SD* = 0.4) prior distribution for the adult LOR population‐level mean parameter. Details on how we derived informative prior distributions are in Appendix 2. We selected logistic (location = 0; scale = 1) prior distributions for all other population‐level mean parameters and gamma (shape = 1; rate = 1) prior distributions for precision parameters *τ_j_*, which provided little prior information.

#### 2.6.3 Renesting rate

We modeled renesting rate as the probability an individual hen would attempt a second nest, conditional upon failure of the first nest, using Bayesian logistic regression. We considered a hen unavailable for renesting if she was killed while tending the first nest. We modeled renesting rate as a function of the age‐class of each hen (adult or yearling), year of the study (2017 or 2018), ordinal date of failure for the previous nest attempt, length of the incubation period for the previous nest attempt (days), and weight of each hen (kg). We used informative prior distributions for the intercept and adult LOR coefficients. Drawing upon the studies in Table 2, we used a Gaussian (mean = −0.7; *SD* = 0.6) prior distribution for the intercept coefficient and a Gaussian (mean = 0.6; *SD* = 0.7) prior distribution for the adult LOR coefficient. Details on how we derived informative prior distributions are in Appendix 2. We selected logistic (location = 0, scale = 1) prior distributions for all other slope coefficients.

#### 2.6.4 Clutch size

We modeled mean clutch size using Bayesian Poisson regression based on the number of eggs laid in each individual nest. Nests were excluded from the analysis if an accurate count of eggs could not be obtained (i.e., the nest was depredated and only some egg fragments remained). We modeled clutch size as a function of age‐class of the nesting hen (adult or yearling), year of the study (2017 or 2018), weight of the nesting hen (kg), and nest attempt (first or second). We used informative prior distributions for the intercept (log expected count when all other slope coefficients equal 0) and the coefficient describing the difference in log expected count between adults and juveniles. Drawing upon the studies in Table 2, we used a Gaussian (mean = 2.4; *SD* = 0.4) prior distribution for the intercept coefficient and a Gaussian (mean = 0.0; *SD* = 0.6) prior distribution for the adult coefficient. Details on how we derived informative prior distributions are in Appendix 2. We selected Gaussian (mean = 0; *SD* = 1) prior distributions for all other slope coefficients.

#### 2.6.5 Hatchability

We modeled hatchability as the proportion of eggs that hatched from each individual nest based on the total number of eggs laid in each individual nest using Bayesian logistic regression. We modeled hatchability as a function of age‐class of the nesting hen (adult or yearling), year of the study (2017 or 2018), and weight of the nesting hen (kg). We used informative prior distributions for the intercept and adult LOR coefficients. Drawing upon the studies in Table 2, we used a Gaussian (mean = 1.3; *SD* = 0.9) prior distribution for the intercept coefficient and a Gaussian (mean = −0.2; *SD* = 1.1) prior distribution for the adult LOR coefficient. Details on how we derived informative prior distributions are in Appendix 2. We selected logistic (location = 0; scale = 1) prior distributions for all other slope coefficients.

#### 2.6.6 Poult survival

We assumed the number of poults alive in each brood *i* at each day posthatch *t* was a Binomial random variable:

$$N_{\mathrm{it}}∼\text{Binomial}\left(\varphi_{\mathrm{it}},N_{i\left(t‐1\right)}\right)$$

where *N_i_*_1_ was equal to the initial number of poults in each brood *i* and *φ_it_* represents daily survival probability of each poult in brood *i* between time *t* − 1 and time *t*. If a brood‐rearing hen died during the 28‐day posthatch interval, we assumed *N_it_* = 0 for all subsequent poult counts. We treated the number of poults counted during each of 3 poult monitoring events at 7, 14, and 28 days posthatch (*t* = 8, 15, 29) as fixed and known, and treated *N_it_* as a latent random variable during all other time steps.

We further assumed a logit‐linear model for daily survival probability which we model as a function of brood age (1–28 days posthatch), age of the brood‐rearing hen (adult or yearling), year of the study (2017 or 2018), precipitation, and temperature. We additionally modeled the interaction between precipitation and temperature. We used an informative prior distribution for the intercept coefficient. Note that we did not include an informative prior distribution for the difference in log odds of poult survival between those raised by adults and juveniles because previous studies (Table 2) did not make this distinction. Drawing upon the studies in Table 2, we used a Gaussian (mean = 3.4; *SD* = 0.1) prior distribution for the intercept coefficient. Details on how we derived informative prior distributions are in Appendix 2. We selected logistic (location = 0; scale = 1) prior distributions for all other slope coefficients.

We fit all models using Bayesian methods to maintain a consistent analytical approach. We fit each model with JAGS version 4.3.0 (Plummer, 2003) via the jagsUI version 1.4.9 interface (Kellner, 2018) in program R version 3.5.1 (R Core Team, 2018). We ran three chains for each model using trace plots to determine an adequate burn‐in period and subsequently ran models until we achieved reasonable convergence ($\hat{R}$ ≤ 1.1; Gelman et al., 2013). We concluded that slope coefficients were different from 0 if 95% credible intervals (CI) did not overlap 0.

## 3 RESULTS

We captured 42 adult and 34 yearling turkey hens during the winter of 2017, and we captured an additional 40 yearling turkey hens during the winter of 2018. Sixteen yearling hens captured during the first year of the study transitioned to the adult age‐class for the second year of the study; twenty‐three adult hens captured during the first year of the study remained in the adult age‐class for the second year of the study. Ultimately, we estimated factors influencing reproductive rates across the 2‐year study from 116 individual turkey hens.

### 3.1 Nesting rate

**FIGURE 3:**
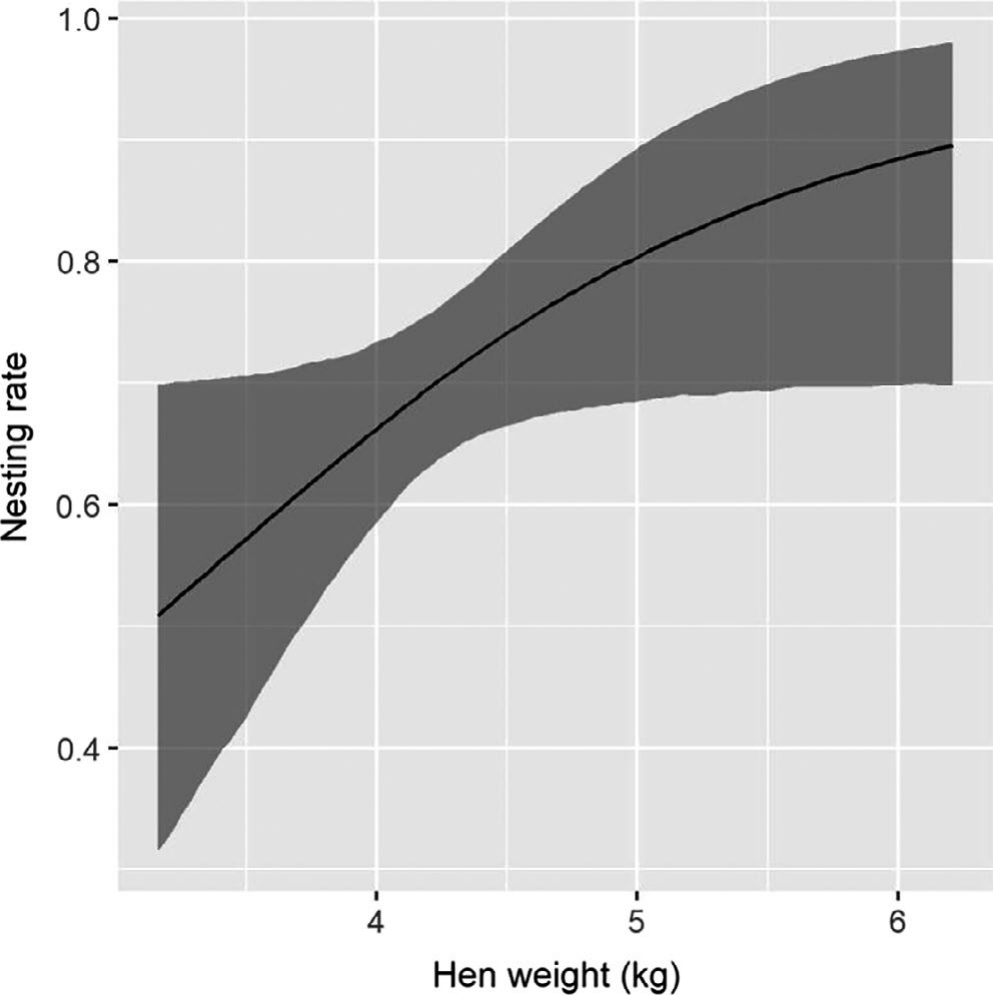
Effect of hen weight (kg) on the probability of nesting for adult eastern wild turkey (*Meleagris gallopavo silvestris*) hens during 2017 and 2018 in northeastern South Dakota, USA

Nesting rate probabilities were estimated from a total of 155 nesting opportunities during 2017 and 2018 (76 hens were available to nest in 2017 and 79 hens were available to nest in 2018; note that 39 hens were available to nest during both years). Our estimate of nesting rate is likely biased low due to our inability to detect nests that were lost during the laying period. Although adult hens had a slightly greater estimated nesting rate (0.82, 95% CI = [0.72, 0.89]) than yearling hens (0.71, 95% CI = [0.65, 0.77]), 95% credible intervals of the age slope coefficient overlapped 0, indicating no strong effect of age. Hen body weight at the time of capture had a positive effect on nesting rate (Figure 3, Table A1). Nesting rate did not differ between years of the study (Table A2).

### 3.2 Nest survival

We observed a total of 147 nest attempts during this study. In 2017, 48 hens attempted one nest, seven hens attempted two nests, and one hen attempted three nests (65 nests total). In 2018, 42 hens attempted one nest, 17 hens attempted two nests, and two hens attempted three nests (82 nests total). We recorded at least one nest attempt in both years from 28 hens. Across both years of the study, five nests were censored due to investigator interference causing the hen to abandon the nest. Five additional nests were censored because we were unable to visit the nest site due to a lack of landowner permissions. Therefore, nest survival probabilities were estimated from a total of 137 nests, across 2,412 days where an individual nest was at risk of failure.

Fifty‐six of 137 nest attempts were successful. Predation of eggs was the leading cause of nest failure, accounting for over half of all failed nest attempts (Table 1). Haying of vegetation surrounding the nest and death of the incubating hen were also major sources of nest failure, accounting for 16% and 12%, respectively, of all failed nest attempts (Table 1). Over a 28‐day average incubation period, estimated survival probability of nests laid by adult hens (0.44, 95% CI = [0.25, 0.62]) was greater than survival probability of nests laid by yearling hens (0.40, 95% CI = [0.20, 0.55]).

**TABLE 1:** Causes of nest failure for nests laid by eastern wild turkey (*Meleagris gallopavo silvestris*) hens during 2017 and 2018 in northeastern South Dakota, USA

Cause of Death	Count	Percentage
Abandoned	7	9%
Death of incubating hen^a^	10	12%
Haying	13	16%
Predation of eggs	47	58%
Trampled by livestock	4	5%
Total	81	

^a^ Nine events caused by predation; 1 event caused by a vehicle collision

**FIGURE 4:**
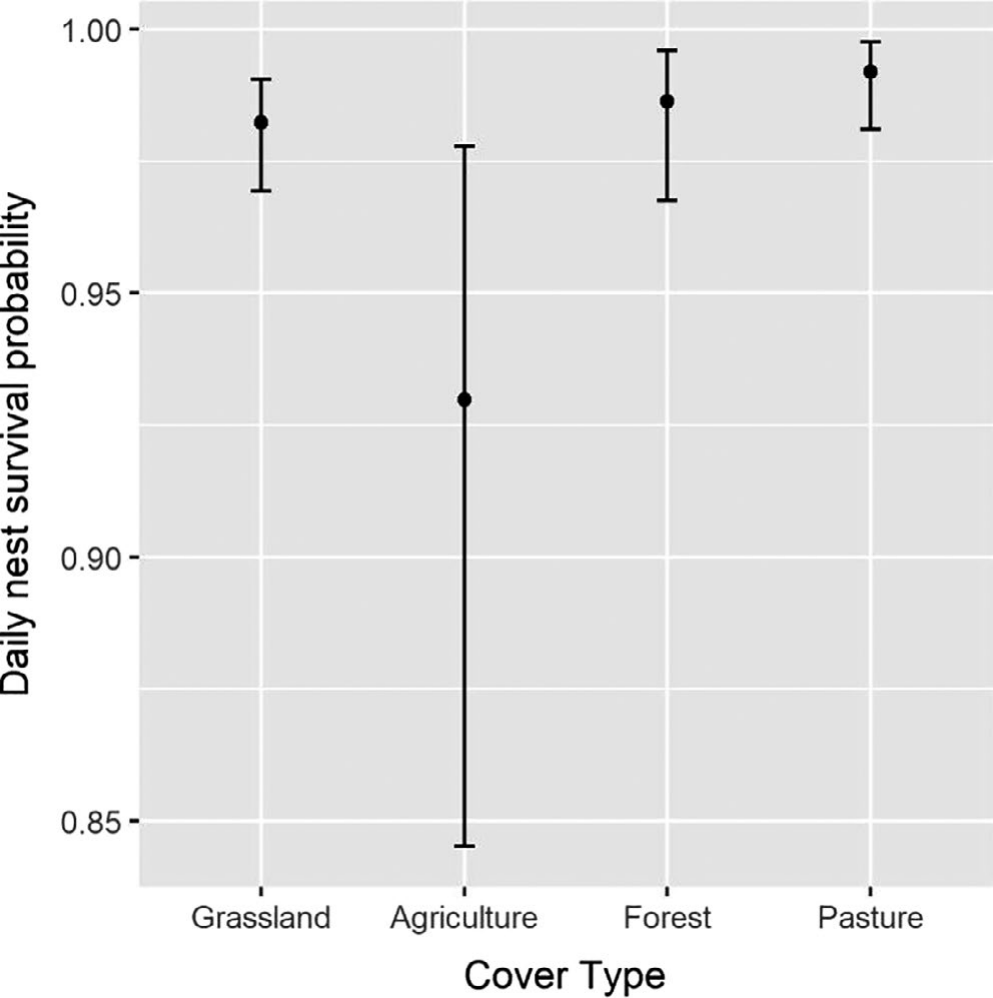
Daily nest survival probability across cover types of adult eastern wild turkey (*Meleagris gallopavo silvestris*) hens nesting in northeastern South Dakota, USA, in 2017 and 2018

**FIGURE 5:**
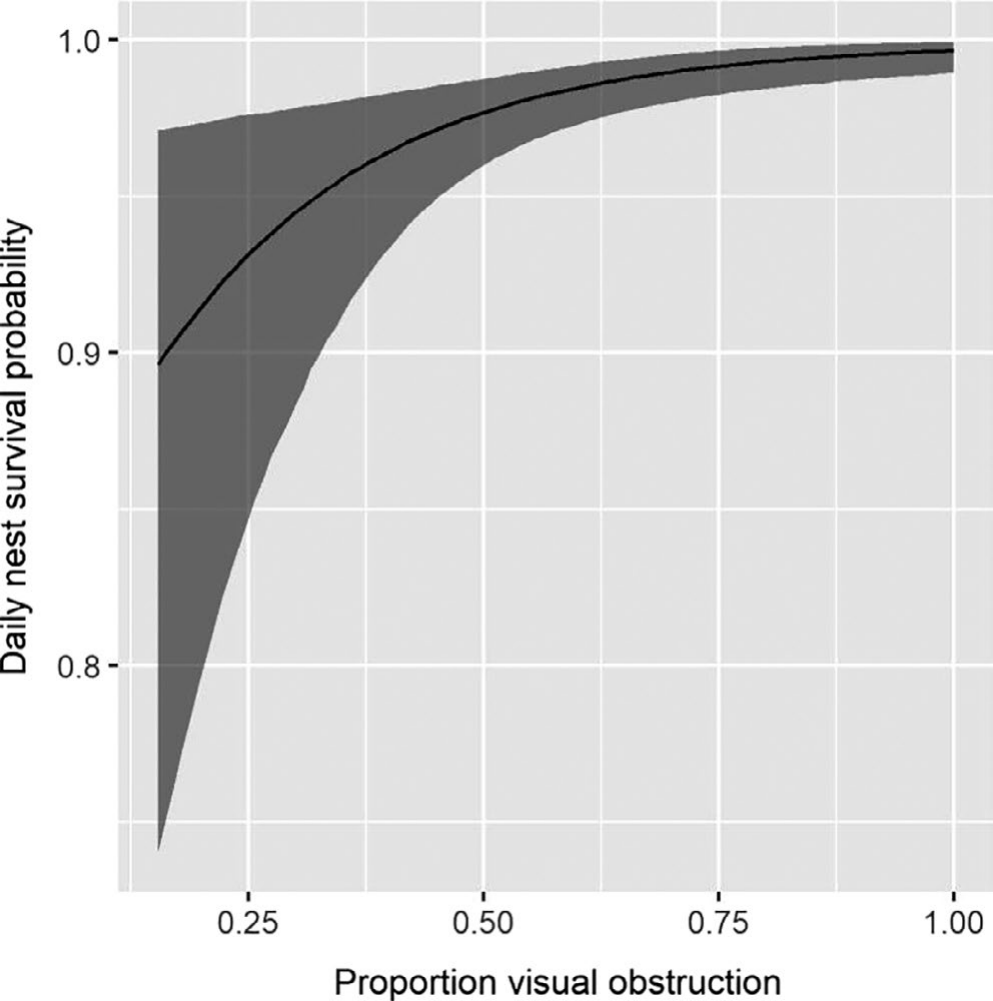
Daily nest survival probability as a function of visual obstruction of adult eastern wild turkey (*Meleagris gallopavo silvestris*) hens nesting in northeastern South Dakota, USA, in 2017 and 2018

**FIGURE 6:**
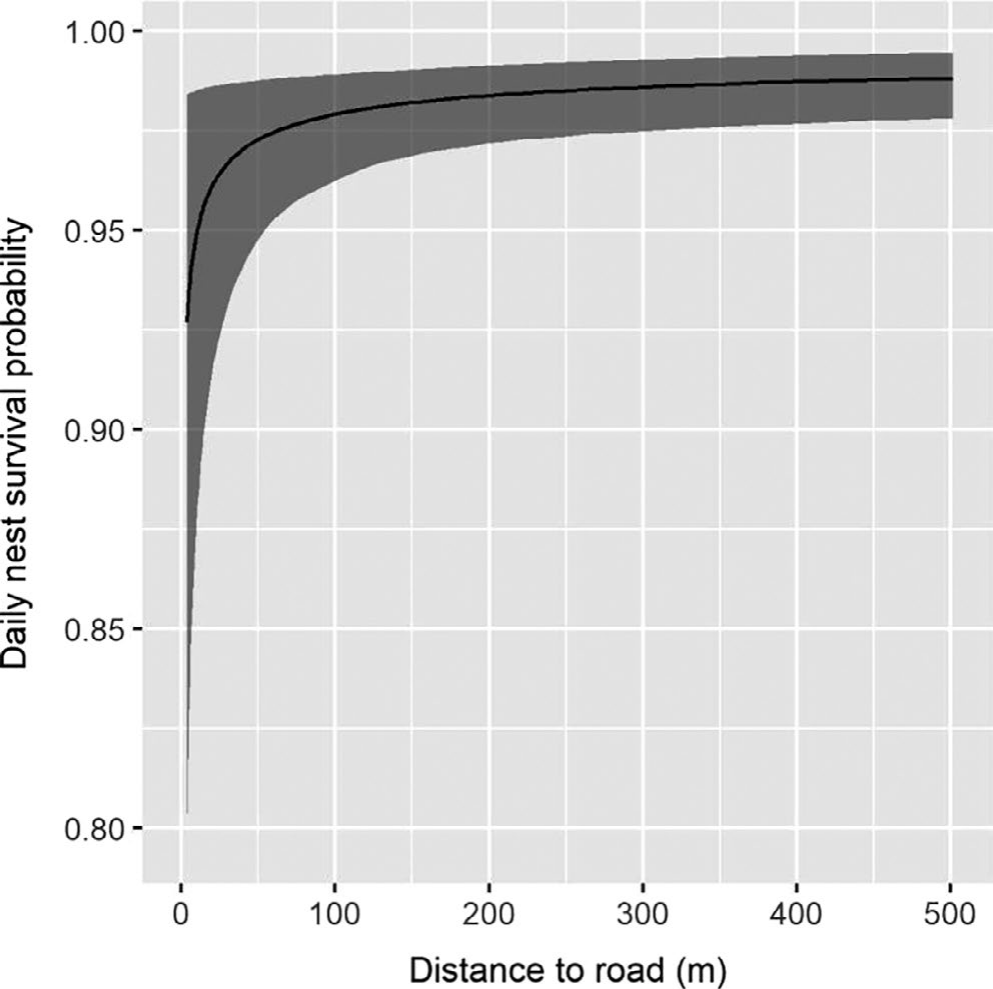
Daily nest survival probability as a function of distance to road of adult eastern wild turkey (*Meleagris gallopavo silvestris*) hens nesting in northeastern South Dakota, USA, in 2017 and 2018

**FIGURE 7:**
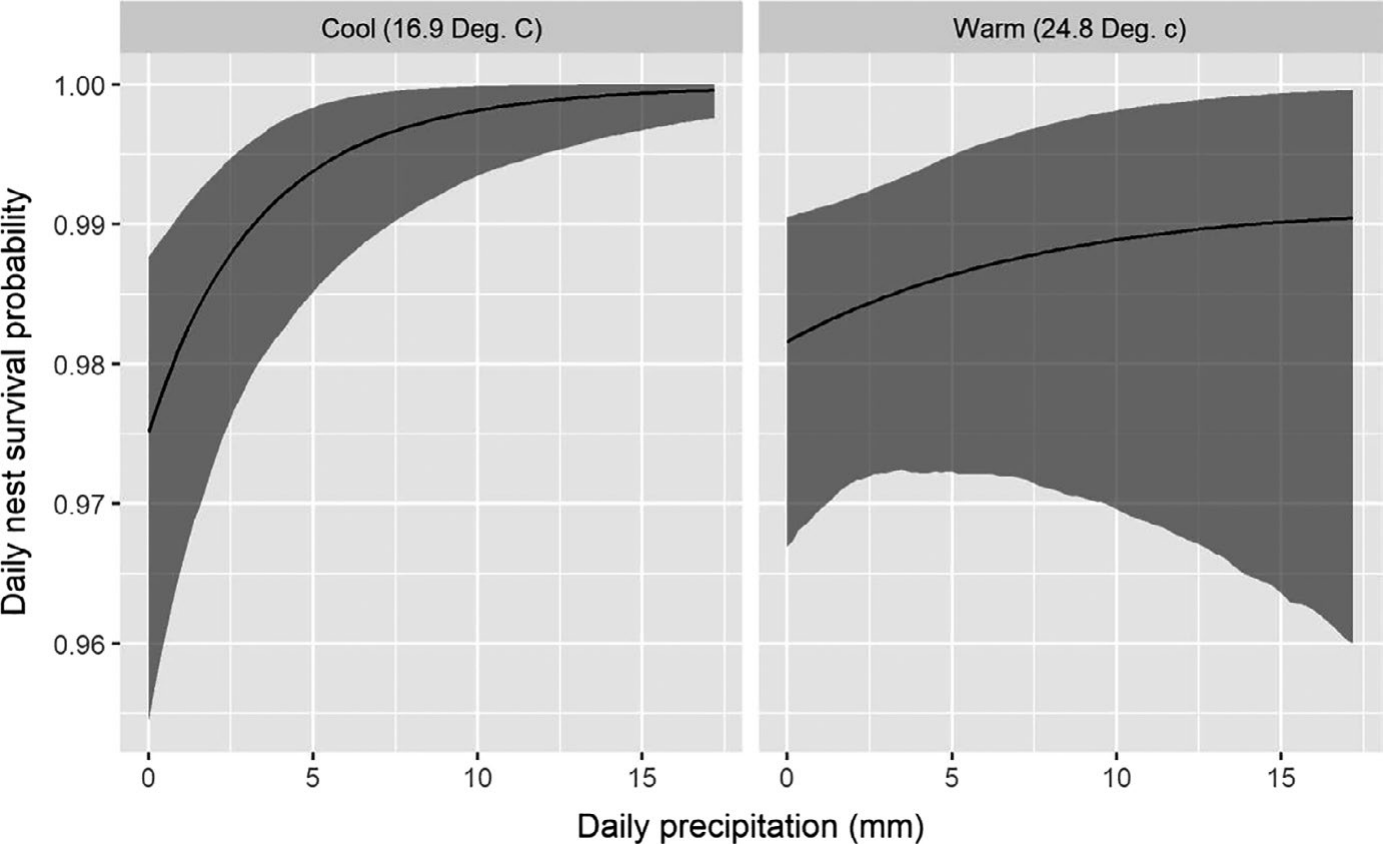
Daily nest survival probability as a function of precipitation and temperature of adult eastern wild turkey (*Meleagris gallopavo silvestris*) hens nesting in northeastern South Dakota, USA, in 2017 and 2018

We found a strong effect of cover type on nest success probability. Nests located in areas classified as agriculture had a much lower success probability relative to nest located in any other cover type (Figure 4, Table A2). All nests that were laid in alfalfa fields failed because fields were hayed (*n* = 10) or depredated (*n* = 2) before eggs hatched. We also found nests with greater visual obstruction (Figure 5, Table A2) and that were placed further from roads (Figure 6, Table A2) had higher daily nest survival probability. Finally, precipitation and temperature had an interactive effect on daily nest survival probability. On relatively cool days, we found a positive effect of precipitation on daily nest survival probability. However, as temperature increased, the effect of precipitation on daily survival diminished. Thus, for a fixed amount of precipitation, predicted daily nest survival tended to be lower on warmer days (Figure 7, Table A2). Daily nest survival probability was not strongly affected by mean total cover (i.e., 95% CI of the population‐level mean overlapped 0; Table A2).

### 3.3 Renesting rate

**FIGURE 8:**
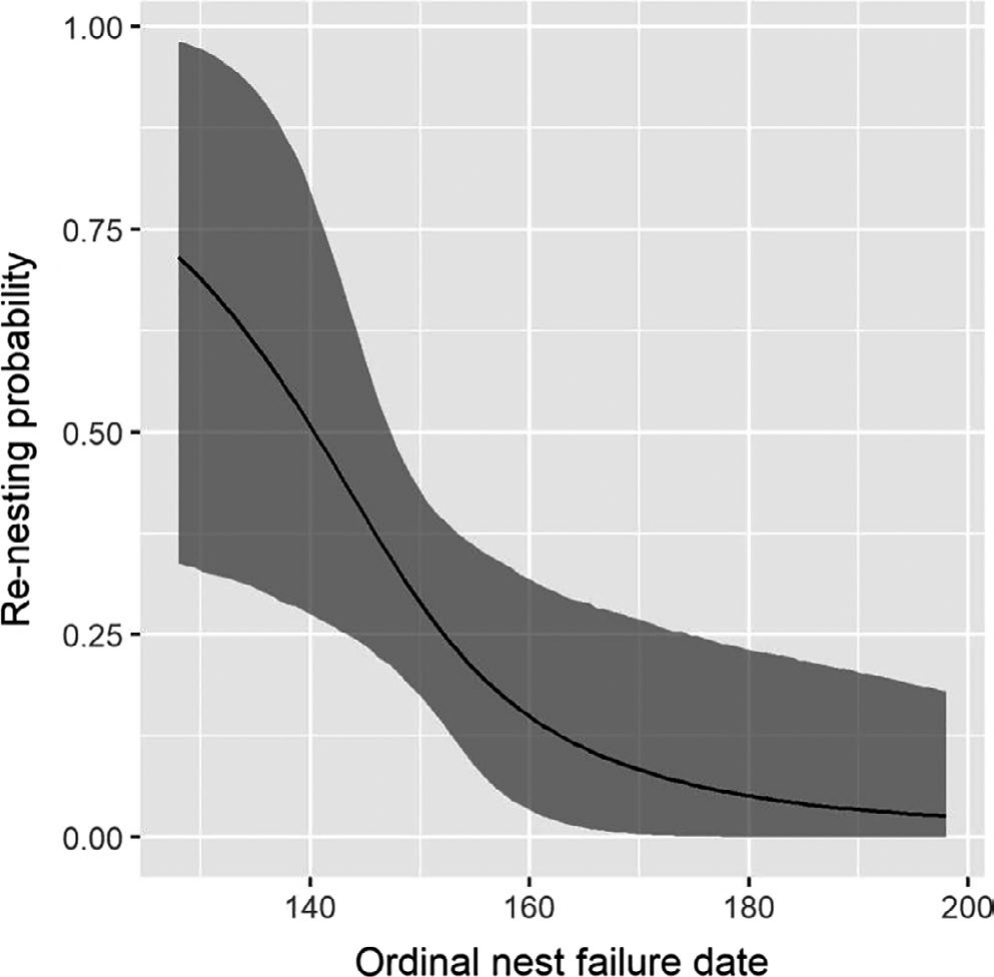
Probability of renesting after a failed nesting attempt as a function of ordinal nest failure date of adult eastern wild turkey (*Meleagris gallopavo silvestris*) hens nesting in northeastern South Dakota, USA, in 2017 and 2018. Ordinal date 160 corresponds to 9 June in both years

Renesting rate probabilities were estimated from a total of 58 hens that were available to renest after a failed first nest attempt (25 in 2017 and 33 in 2018). Adult hens were more likely to renest (0.59, 95% CI = [0.41, 0.76]) than yearling hens (0.26, 95% CI = [0.14, 0.40]). Probability of renesting was lower when the date of nest failure for the previous nest attempt was later in the season (Figure 8, Table A3). Renesting rate did not differ between years of the study and was not affected by hen weight at the time of capture or duration of previous nesting attempt (Table A3).

### 3.4 Clutch size

Clutch size was estimated from a sample size of 105 nests (48 nests in 2017 and 57 nests in 2018). We were unable to determine accurate clutch counts for 40 out of 91 failed nest attempts and two out of 56 successful nest attempts over the course of this study, and therefore, omitted these nests from the clutch size analysis. During the first nest attempt, the mean clutch size laid by adult hens was 10.6 (95% CI = [9.8, 11.5]), the mean clutch size laid by yearling hens was 10.0 (95% CI = [8.9, 11.2]), and clutch size did not vary by age‐class. Clutch size did not differ between nesting attempts or years of the study and was not affected by hen body weight at the time of capture (Table A4).

### 3.5 Hatchability

Hatchability was estimated from a sample size of 54 successful nests (25 nests in 2017 and 29 nests in 2018; seven hens successfully hatched a clutch during both years of the study). Hatchability of clutches laid by adult hens (0.88, 95% CI = [0.85, 0.92]) was not different from clutches laid by yearling hens (0.87, 95% CI = [0.82, 0.92]). Hatchability did not differ between years of the study and was not affected by hen body weight (kg) at the time of capture (Table A5).

### 3.6 Poult survival

**FIGURE 9:**
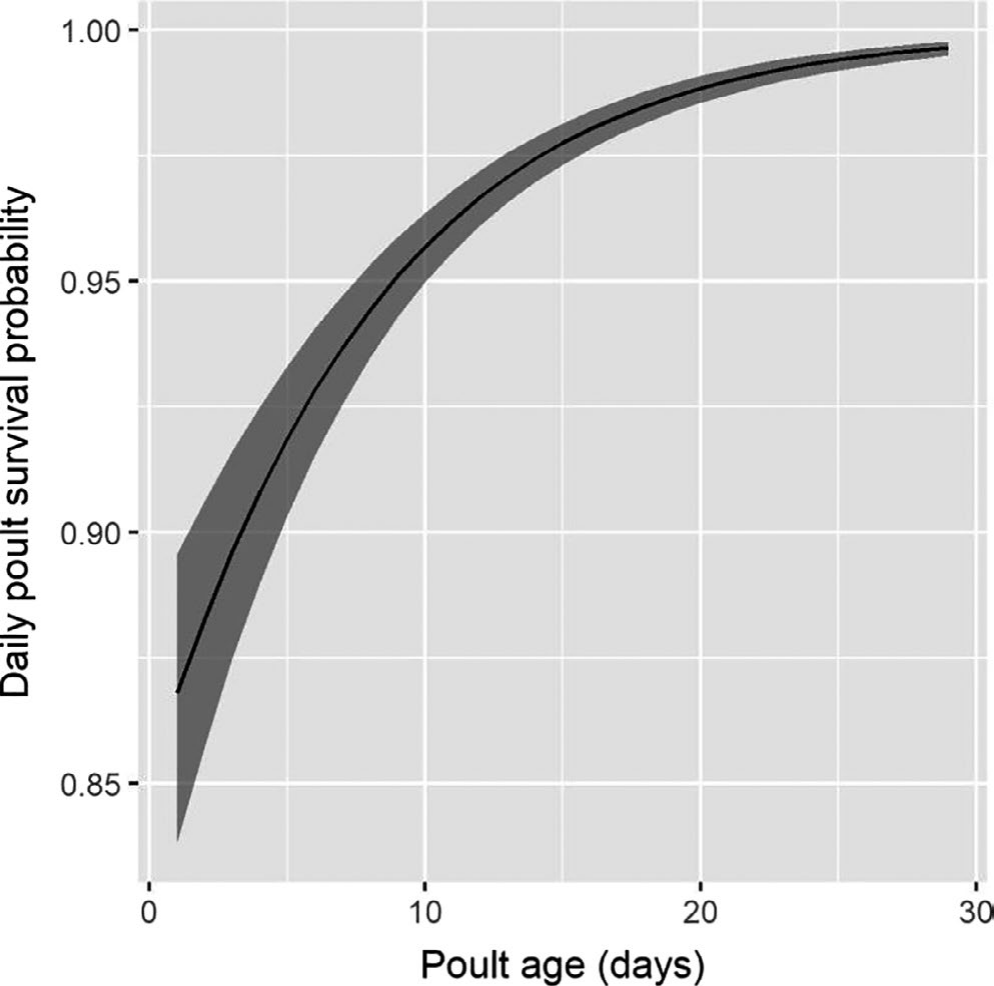
Daily survival probability of eastern wild turkey (*Meleagris gallopavo silvestris*) poults as function of age in northeastern South Dakota, 2017 and 2018

Poult survival probability was estimated from a total of 55 broods (26 broods in 2017 and 29 broods in 2018; seven hens had broods in both years). Poults reared by adult hens had a greater probability of surviving the 28‐day posthatch interval (0.33, 95% CI = [0.28, 0.38]) than poults reared by yearlings (0.16, 95% CI = [0.11, 0.21]). Daily poult survival probability increased as the number of days posthatch increased (i.e., as poults become older; Figure 9, Table A6). Daily poult survival probability was not affected by precipitation or temperature and did not differ between years of the study (Table A6).

## 4 DISCUSSION

Our investigation of a turkey population exhibiting an apparent decline allowed us to identify important sources of variation in reproductive rates and identify factors potentially contributing to the apparent decline. Many of our estimates of reproductive rates—nest survival, renesting rate, clutch size, and hatchability—were well within the range of previously reported estimates for these reproductive rates (Table 2). However, adult hen nesting rate and the probability of poults raised by yearlings surviving to 28 days were low when compared to other studies (Lehman et al., 2001; Pollentier, Lutz, et al., 2014; Shields & Flake, 2006) (Table 2). Poult survival to 28 days posthatch is an important demographic rate affecting turkey population dynamics (Hubbard, Garner, & Klaas, 1999; Pollentier, Hull, et al., 2014; Vangilder & Kurzejeski, 1995), and the low survival probability of poults raised by yearlings could be contributing to decreased productivity of hens in northeastern South Dakota and the apparent population decline.

**TABLE 2:** Comparison of estimates of eastern wild turkey (*Meleagris gallopavo silvestris*) reproductive rates obtained from northeastern South Dakota in 2017 and 2018 with previously published estimates

Parameter	This Study		Published			Reference
	Adult	Yearling	Adult	Yearling	Combined	
Nesting rate	0.82	0.71	0.90 (0.03)	0.34 (0.09)	‐	Pollentier, Lutz, et al., 2014
			0.94 (0.03)	0.91 (0.06)	‐	Shields & Flake, 2006
			0.96	0.88	‐	Porter et al., 1983
			0.98 (0.01)	0.79 (0.06)	‐	Paisley et al., 1998
			1	0.81	‐	Vander Haegen et al., 1988
			‐	‐	0.97	Vangilder & Kurzejeski, 1995
			‐	‐	1.00	Vangilder, Kurzejeski, Kimmel‐Truitt, & Lewis, 1987
Nest survival	0.44	0.40	0.16 (0.03)	0.05 (0.03)	‐	Paisley et al., 1998
			0.23 (0.06)	0.12 (0.13)	‐	Pollentier, Lutz, et al., 2014
			0.35 (0.09)	0.20 (0.04)	‐	Vangilder & Kurzejeski, 1995
			‐	‐	0.31	Vangilder et al., 1987
			0.51 (0.05)	0.47 (0.11)	‐	Shields & Flake, 2006
			0.64	0.61	‐	Porter et al., 1983
			0.68	0.33	‐	Vander Haegen et al., 1988
Renesting rate	0.59	0.26	0.51 (0.08)	0.22 (0.14)	‐	Shields & Flake, 2006
			0.39 (0.08)	0.46 (0.08)	‐	Vangilder & Kurzejeski, 1995
			0.41 (0.09)	0.00 (0.00)	‐	Pollentier, Lutz, et al., 2014
			‐	‐	0.5	Vander Haegen et al., 1988
			‐	‐	0.55	Vangilder et al., 1987
			0.60 (0.05)	0.42 (0.08)	‐	Paisley et al., 1998
			‐	‐	0.65	Porter et al., 1983
Clutch size	10.6	10.0	‐	‐	10.03 (0.24)	Vangilder et al., 1987
			‐	‐	10.38 (0.21)	Vangilder & Kurzejeski, 1995
			‐	‐	10.67 (0.33)	Pollentier, Lutz, et al., 2014
			11.2 (0.36)	10.3 (0.48)	‐	Paisley et al., 1998
			11.7	12.8	‐	Vander Haegen et al., 1988
			12.8 (1.9)	11.1 (1.9)	‐	Porter et al., 1983
Hatchability	0.88	0.87	0.76 (0.28)	0.83 (0.21)	‐	Porter et al., 1983
			0.83 (0.07)	0.86 (0.06)	‐	Vander Haegen et al., 1988
			‐	‐	0.87 (0.04)	Paisley et al., 1998
			‐	‐	0.90 (0.03)	Vangilder & Kurzejeski, 1995
			‐	‐	0.92 (0.03)	Pollentier, Lutz, et al., 2014
Poult survival	0.33	0.16	‐	‐	0.36 (0.05)	Shields & Flake, 2006
			‐	‐	0.37 (0.06)	Pollentier, Lutz, et al., 2014
			‐	‐	0.381	Vangilder et al., 1987
			‐	‐	0.40 (0.15)	Hubbard et al., 1999
			‐	‐	0.41	Roberts & Porter, 1998
			‐	‐	0.45	Vangilder & Kurzejeski, 1995
			‐	‐	0.47 (0.12)	Paisley et al., 1998

Note

Published estimates are distinguished by age (adult or yearling) when possible or reported as “combined” if the authors did not distinguish estimates by age. Standard errors of published point estimates are presented in parentheses when reported by the authors.

Although we found nest survival probability was well within the range of published estimates, we identified important sources of variation in this reproductive rate. In particular, we found that nest survival was substantially lower in agricultural fields relative to other cover types. This was largely driven by failure of all nests placed in alfalfa fields, which were mostly lost due to haying operations. Haying can be an important cause of mortality and nest failure for turkey and other avian species within agricultural landscapes (Bollinger, Bollinger, & Gavin, 1990; Shields & Flake, 2006; Wright, Paisley, & Kubisiak, 1996). Standard recommendations for wildlife‐friendly farming include delaying haying until mid‐July to allow nests in agricultural fields to fledge, haying from the center of the field and working out to allow nesting birds to escape, and installing flushing bars to minimize mortality (NRCS, 1999). Incentive programs designed to restrict haying during grassland bird nesting seasons have been demonstrated to improve avian reproductive success (Perlut, Strong, & Alexander, 2011). However, delaying haying operations can come at high economic costs to farmers and such wildlife‐friendly practices are unlikely to succeed without established incentive programs.

Like the agricultural activities describe above, predation is another important source of nest failure. Nest‐site selection is strongly driven by avoidance of predation (Gill, 2007). Turkeys are known to select nests sites that are visually obstructed to predators (Isabelle, Conway, Comer, Calkins, & Hardin, 2016; Wood, Cohen, Conner, Collier, & Chamberlain, 2019), and our study demonstrated that selection for nest sites with high visual obstruction may reduce predation risk, as the probability of nest success was greater at sites with relatively high visual obstruction (Yarnall, Litt, & Lehman, 2019). Turkeys may also select nests located farther away from roads as a predator avoidance strategy. Beasley, DeVault, and Rhodes (2007) noted high raccoon prevalence near roads, and coyotes are known to use roads as travel corridors and forage along roads (Tigas, Van Vuren, & Sauvajot, 2002; Gosselink, Van Deelen, Warner & Joselyn, 2003; Hinton, van Manen, & Chamberlain, 2015). Our study found that nests located close to roads had a lower probability of survival, likely due to increased predation risk near roads. This result is similar to Badyaev (1995), who found that successful turkey nests were located farther from roads, on average, than unsuccessful nests.

While many predators hunt visually, many also hunt by olfaction. Roberts and Porter (1998b) and Lehman, Rumble, et al. (2008) found a negative association between precipitation and nest survival probability, presumably because of increased detection of incubating hens by predators. Our finding of a positive association with daily nest survival probability and amount of precipitation at cold temperatures seemingly contradicts these findings. However, we found that for a given amount of precipitation, survival probability decreased as temperature increased, which is consistent with Roberts and Porter's (1998b) suggestion that olfactory cues to locate nests may be enhanced during warm, wet periods. Alternatively, the nesting seasons captured during this study were, on average, drier than a typical spring in our study area (Figure 2). It is possible that the effects of precipitation on nest survival may extend beyond individual rainfall events and be cumulative in nature, such that rainfall events in relatively dry years may influence nest survival differently than rainfall events in relatively wet years.

In addition to external factors such as cover type, temperature, and precipitation, we found intrinsic factors such as experience and body condition were important sources of variation in reproductive rates. For example, nesting rate increased as hen body weight increased, likely because heavier birds are better able to complete the energetically expensive task of incubating a clutch (Porter et al., 1983). Following nest failure, adult hens were more likely to renest than yearling hens, which is consistent with previous research (Lehman et al., 2001; Pollentier, Lutz, et al., 2014; Shields & Flake, 2006). Similarly, poults reared by adult hens had a greater daily survival than poults reared by yearling hens, consistent with studies of Merriam's turkey in the Black Hills, South Dakota (Lehman, Flake, et al., 2008). Finally, poult survival probability increased with the number of days posthatch, which is also consistent with many other studies (Hubbard et al., 1999; Lehman et al., 2001; Paisley et al., 1998; Pollentier, Lutz, et al., 2014; Porter et al., 1983; Shields & Flake, 2006; Switzer & Tucker, 2009; Vander Haegen et al., 1988; Vangilder & Kurzejeski, 1995). Older poults are better able to escape ground predators (Spears et al., 2007) and thermoregulate (Healy & Nenno, 1985; Schmidt‐Nielsen, 1997), increasing daily survival probability as they age.

An important source of uncertainty in the apparent regional decline in turkey abundance is the role of loss of land enrolled in the Conservation Reserve Program (CRP). The amount of land enrolled in CRP has been steadily declining for more than a decade (Hellerstein, 2017), with some of the steepest declines occurring in northeastern South Dakota (USDA, 2016). It is possible that conversion of former CRP lands to agricultural production could translate to increased probability of nesting in agricultural fields, leading to decreases in reproductive output and consequently declining population growth rates. Future research should determine how nest‐site selection varies as a consequence of loss of CRP habitat, and whether loss of CRP habitat increases the probability of nesting in low productivity habitats such as agricultural fields.

Our research highlighted reproductive parameters that may be contributing to an apparent population decline in turkey in northeastern South Dakota. Survival of poults raised by yearlings is low compared to other published studies. However, since uncontrollable intrinsic (e.g., hen age) and extrinsic (e.g., precipitation) factors appeared to have the greatest effects on poult survival, there is little managers can do to increase poult survival, particularly without knowledge of factors contributing to low survival. Of all the reproductive parameters we evaluated, nest survival may be the most amenable to changes through management action. Although our estimates of nest survival were not low compared to other studies, neither were the estimates close to upper limits observed in other studies and there may be room to increase nest survival and reproductive output. In particular, we found low daily survival probability in agricultural fields relative to other cover types. To increase nest survival in these cover types, managers could promote wildlife‐friendly practices such as delayed haying (NRCS, 1999). Alternatively, increasing the availability of suitable nesting cover types (e.g., CRP fields) may lead to lower probabilities of nesting in agricultural fields. Improvements to productivity and recruitment could potentially stabilize, or lead to growth of, the population of turkeys in northeastern South Dakota.

## CONFLICT OF INTEREST

The authors have declared that no competing interests exist.

## AUTHOR CONTRIBUTIONS

**Reina M. Tyl:** Data curation (lead); formal analysis (equal); investigation (lead); methodology (equal); visualization (equal); writing – original draft (lead); writing – review and editing (equal). **Christopher T. Rota:** Conceptualization (equal); formal analysis (equal); funding acquisition (equal); methodology (equal); project administration (equal); resources (equal); software (equal); supervision (equal); visualization (equal); writing – original draft (equal); writing – review and editing (equal). **Chadwick P. Lehman:** Conceptualization (equal); funding acquisition (equal); methodology (equal); project administration (equal); resources (equal); supervision (equal); writing – review and editing (equal).

### Open Research Badges

This article has earned an Open Data Badge for making publicly available the digitally‐shareable data necessary to reproduce the reported results. The data is available at https://doi.org/10.5061/dryad.5dv41ns3d.

## Data Availability

Data are archived in the Dryad Data Repository. Link to data during review process: https://datadryad.org/stash/share/0SbTuPgkKGU6H0TBuzd3yLDsHeGb50oCZrlcFwgTZ3s and DOI upon data publication: https://doi.org/10.5061/dryad.5dv41ns3d.

## APPENDIX 1

**TABLE A1:** Log odds ratio (LOR), lower 95% credible interval (CI) level, and upper 95% CI level for each slope coefficient obtained from Bayesian nesting rate model fit to data collected in 2017 and 2018 from eastern wild turkeys (*Meleagris gallopavo silvestris*) in northeastern South Dakota, USA

Covariate	LOR	Lower CI level	Upper CI level
(Intercept)	0.92	0.62	1.25
Hen age: adult	0.59	−0.03	1.24
Study year: 2018^a^	0.16	−0.23	0.53
Hen weight	0.45	0.00	0.90

^a^ Note, we used sum‐to‐zero constraints for study year. Therefore, the coefficient for study year 2017 = −1 × the coefficient reported above.

**TABLE A2:** Population‐level mean log odds ratio (LOR), lower 95% credible interval (CI) level, and upper 95% CI level for each slope coefficient obtained from Bayesian nest survival model fit to data collected in 2017 and 2018 from eastern wild turkeys (*Meleagris gallopavo silvestris*) in northeastern South Dakota, USA

Covariate	LOR	Lower CI Level	Upper CI Level
(Intercept)	3.49	3.07	3.91
Hen age: adult	0.60	0.08	1.15
Precipitation	1.07	0.43	1.97
Temperature	0.05	−0.32	0.41
Precipitation × temperature	−0.85	−1.59	−0.21
Cover type: agriculture	−1.35	−2.44	−0.28
Cover type: forest	0.30	−0.71	1.36
Cover type: pasture	0.85	−0.19	1.96
Mean total cover	−0.32	−0.89	0.25
Mean VOR	0.89	0.32	1.45
Distance to road	0.48	0.03	0.95

**TABLE A3:** Log odds ratio (LOR), lower 95% credible interval (CI) level, and upper 95% CI level for each slope coefficient obtained from Bayesian renesting rate model fit to data collected in 2017 and 2018 from eastern wild turkeys (*Meleagris gallopavo silvestris*) in northeastern South Dakota, USA

Covariate	LOR	Lower CI level	Upper CI level
(Intercept)	−1.10	−1.76	−0.41
Hen age: adult	1.49	0.57	2.38
Study year: 2018^a^	0.61	−0.00	1.28
Hen weight	0.03	−0.65	0.74
Previous nest duration	−0.02	−0.75	0.71
Previous fail date	−0.96	−1.81	−0.20

^a^ Note, we used sum‐to‐zero constraints for study year. Therefore, the coefficient for study year 2017 = −1 × the coefficient reported above.

**TABLE A4:** Log proportional change, lower 95% credible interval (CI) level, and upper 95% CI level for each slope coefficient obtained from Bayesian clutch size model fit to data collected in 2017 and 2018 from eastern wild turkeys (*Meleagris gallopavo silvestris*) in northeastern South Dakota, USA

Covariate	LOR	Lower CI level	Upper CI level
(Intercept)	2.31	2.18	2.42
Hen age: adult	0.05	−0.08	0.19
Study year: 2018^a^	−0.02	−0.08	0.04
Hen weight	0.03	−0.04	0.09
Nest attempt: renest^a^	0.00	−0.07	0.07

^a^ Note, we used sum‐to‐zero constraints. Therefore, the coefficient for the second category (year 2017 or nest attempt 1) = −1 × the coefficient reported above.

**TABLE A5:** Log odds ratio (LOR), lower 95% credible interval (CI) level, and upper 95% CI level for each slope coefficient obtained from Bayesian hatchability model fit to data collected in 2017 and 2018 from eastern wild turkeys (*Meleagris gallopavo silvestris*) in northeastern South Dakota, USA

Covariate	LOR	Lower CI level	Upper CI level
(Intercept)	1.97	1.52	2.46
Hen age: adult	0.07	−0.53	0.66
Study year: 2018^a^	−0.09	−0.36	0.17
Hen weight	−0.01	−0.30	0.30

^a^ Note, we used sum‐to‐zero constraints for study year. Therefore, the coefficient for study year 2017 = −1 × the coefficient reported above.

**TABLE A6:** Log odds ratio (LOR), lower 95% credible interval (CI) level, and upper 95% CI level for each slope coefficient obtained from Bayesian poult survival model fit to data collected in 2017 and 2018 from eastern wild turkeys (*Meleagris gallopavo silvestris*) in northeastern South Dakota, USA

Covariate	LOR	Lower CI level	Upper CI level
(Intercept)	3.53	3.38	3.69
Poult age	1.13	0.97	1.29
Hen age: adult	0.25	0.13	0.36
Study year: 2018	−0.10	−0.32	0.11
Precipitation	0.34	−0.11	1.09
Temperature	−0.13	−0.40	0.09
Precipitation × temperature	−0.03	−1.11	0.62

## APPENDIX 2

Details regarding how informative prior distributions were obtained.

### NESTING RATE, RENESTING RATE, AND HATCHABILITY

We derived informative prior distributions for the intercept and adult log odds ratio (LOR) coefficients by drawing on those studies in Table 2 that distinguish between adult and juvenile nesting rate, renesting rate, and hatchability. For each reproductive parameter, we integrated previous studies into a beta‐binomial model:

$$y_{i}∼\text{Binomial}\left(p_{i},N_{i}\right)$$

$$p_{i}∼\text{Beta}\left(a,b\right)$$

where *y_i_* is the number of “successes” from study *i* (defined as hens nesting, hens renesting, or eggs hatching), *N_i_* is the total number of “trials” included in study *i* (hens available to nest, hens available to renest, or eggs laid), *p_i_* is the probability of success, and *a* and *b* are shape parameters governing the beta distribution. We assumed gamma (shape = 0.1; rate = 0.1) prior distributions for both *a* and *b*. For each reproductive parameter, we separately estimated posterior distributions of *a* and *b* for both adults and juveniles.

After obtaining posterior distributions of *a* and *b* for both adults and juveniles, we estimated the posterior distribution of the probability of success as:

$$r=\frac{a}{a+b}$$

which is the expected value of a beta random variable. Since we modeled the probability of success for each reproductive parameter on the logit‐linear scale, we transformed the quantity *r* (which is bound between 0 and 1) to the log odds scale:

s=log(r)‐log(1‐r),

which is the well‐known logit transformation. To approximate the adult LOR coefficient, we calculated the difference in *s* obtained from juvenile and adult log odds of success:

$$t=s_{a}‐s_{j}$$

where *s_a_* and *s_j_* are posterior distributions of *s* obtained from adults and juveniles, respectively.

Distributions of both *s* and *t* were very well approximated with a Gaussian distribution. We therefore used Gaussian distributions as prior distributions for the intercept and adult LOR coefficients. We used a Gaussian prior distribution with mean and *SD* calculated from posterior samples of *s_j_* for the intercept coefficient of our logit‐linear models of nesting probability, renesting probability, and hatchability. We used a Gaussian prior distribution with mean and *SD* calculated from posterior samples of *t* for the adult LOR slope coefficient of our logit‐linear models of nesting probability, renesting probability, and hatchability.

### NEST SURVIVAL

We derived informative prior distributions for the intercept and adult log odds ratio (LOR) coefficients by drawing on those studies in Table 2 that distinguish between adult and juvenile nest success. We assumed the probability that a hen successfully hatched at least 1 poult reported from study *i* was a beta random variable:

$$p_{i}∼\text{Beta}\left(a,b\right)$$

where *a* and *b* are shape parameters governing the beta distribution. We assumed gamma (shape = 0.1; rate = 0.1) prior distributions for both *a* and *b*. We separately estimated posterior distributions of *a* and *b* for both adults and juveniles.

After obtaining posterior distributions of *a* and *b* for both adults and juveniles, we estimated the posterior distribution of a hen successfully hatching at least 1 poult as:

$$r=\frac{a}{a+b}$$

which is the expected value of a beta random variable. Since we modeled the daily probability of nest survival on the logit‐linear scale, we transformed the quantity *r* (which is bound between 0 and 1) to the log odds scale:

$$s=\log\left(r^{1/28}\right)‐\log\left(1‐r^{1/28}\right),$$

which is the well‐known logit transformation. Note that we also transformed the probability of hatching at least 1 poult to daily nest survival probability by taking the 28th root of *r*, to reflect a 28‐day incubation period. To approximate the adult LOR coefficient, we calculated the difference in *s* obtained from juvenile and adult log odds of daily nest survival:

$$t=s_{a}‐s_{j}$$

where *s_a_* and *s_j_* are posterior distributions of *s* obtained from adults and juveniles, respectively.

Distributions of both *s* and *t* were very well approximated with a Gaussian distribution. We therefore used Gaussian distributions as prior distributions for the intercept and adult LOR coefficients. We used a Gaussian prior distribution with mean and *SD* calculated from posterior samples of *s_j_* for the intercept coefficient of our logit‐linear models of daily nest survival. We used a Gaussian prior distribution with mean and *SD* calculated from posterior samples of *t* for the adult LOR slope coefficient of our logit‐linear models of daily nest survival.

### POULT SURVIVAL

We derived informative prior distributions for the intercept coefficient by drawing on those studies in Table 2 that report the probability of poult survival to 28 days. We assumed the probability that a poult survived to 28 days reported from study *i* was a beta random variable:

$$p_{i}∼\text{Beta}\left(a,b\right)$$

where *a* and *b* are shape parameters governing the beta distribution. We assumed gamma (shape = 0.1; rate = 0.1) prior distributions for both *a* and *b*. We did not distinguish between poults raised by adults or yearlings, as this distinction was not made in studies listed in Table 2.

After obtaining posterior distributions of *a* and *b*, we estimated the posterior distribution of a poult surviving to 28 days as:

$$r=\frac{a}{a+b}$$

which is the expected value of a beta random variable. Since we modeled the daily probability of poult survival on the logit‐linear scale, we transformed the quantity *r* (which is bound between 0 and 1) to the log odds scale:

$$s=log\left(r^{1/28}\right)‐log\left(1‐r^{1/28}\right),$$

which is the well‐known logit transformation. Note that we also transformed the probability of a poult surviving to 28 days to daily poult survival probability by taking the 28th root of *r*. The distribution of *s* was very well approximated with a Gaussian distribution. We therefore used a Gaussian prior distribution with mean and *SD* calculated from posterior samples of *s* for the intercept coefficient of our logit‐linear model of daily poult survival.

### CLUTCH SIZE

We derived informative prior distributions for the intercept and difference in log expected count between adults and juveniles (hereafter simply “adult”) coefficients by drawing on those studies in Table 2 that distinguish between adult and juvenile clutch size. We assume mean clutch size reported from study *i* was a gamma random variable:

$$p_{i}∼\text{Gamma}\left(a,b\right)$$

where *a* and *b* are shape and rate parameters, respectively, governing the gamma distribution. We assumed gamma (shape = 1, rate = 1) prior distributions for both *a* and *b*. We separately estimated posterior distributions of *a* and *b* for both adults and juveniles.

After obtaining posterior distributions of *a* and *b* for both adults and juveniles, we estimated the posterior distribution of expected clutch size as:

$$r=\frac{a}{b}$$

which is the expected value of a gamma random variable. Since we modeled expected clutch size on the log scale, we transformed the quantity *r* as:

s=log(r).

To approximate the adult coefficient, we calculated the difference in *s* obtained from juvenile and adult clutch size models:

$$t=s_{a}‐s_{j}$$

where *s_a_* and *s_j_* are posterior distributions of *s* obtained from adults and juveniles, respectively.

Distributions of both *s* and *t* were very well approximated with a Gaussian distribution. We therefore used Gaussian distributions as prior distributions for the intercept and adult coefficients. We used a Gaussian prior distribution with mean and *SD* calculated from posterior samples of *s_j_* for the intercept coefficient of our log‐linear model of clutch size. We used a Gaussian prior distribution with mean and *SD* calculated from posterior samples of *t* for the adult coefficient of our log‐linear model of clutch size.

## References

[ece36583-bib-0001] Badyaev, A. V. (1995). Nesting habitat and nesting success of eastern wild turkeys in the Arkansas Ozark highlands. The Condor, 97, 221–232. 10.2307/1368998

[ece36583-bib-0002] Beasley, J. C. , Devault, T. L. , & Rhodes, O. E. Jr (2007). Home‐range attributes of raccoons in a fragmented agricultural region of northern Indiana. The Journal of wildlife management, 71(3), 844–850.

[ece36583-bib-0003] Benkobi, L. , Uresk, D. W. , Schenbeck, G. , & King, R. M. (2000). Protocol for monitoring standing crop in grasslands using visual obstruction. Journal of Range Management, 53, 627–633. 10.2307/4003158

[ece36583-bib-0004] Bollinger, E. K. , Bollinger, P. B. , & Gavin, T. A. (1990). Effects of hay‐cropping on eastern populations of the bobolink. Wildlife Society Bulletin, 111, 142–150.

[ece36583-bib-0005] Caswell, H. (2001). Matrix population models. Sunderland, MA: Sinauer Associates Inc.

[ece36583-bib-0006] Clawson, M. R. , & Rotella, J. J. (1998). Success of artificial nests in CRP fields, native vegetation, and field borders in southwestern Montana. Journal of Field Ornithology, 69, 180–191.

[ece36583-bib-0007] Daubenmire, R. F. (1959). Canopy coverage method of vegetation analysis. Northwest Science, 33, 43–64.

[ece36583-bib-0008] Fair, J. M. , Paul, E. , & Jones, J. (2010). Guidelines to the use of wild birds in research. Washington, DC: Ornithological Council.

[ece36583-bib-0009] Flint, R. F. (1955). Pleistocene Geology of Eastern South Dakota. Washington, DC: Government Printing Office.

[ece36583-bib-0010] Gelman, A. , & Hill, J. (2007). Data analysis using regression and multilevel/hierarchical models. New York, NY: Cambridge University Press.

[ece36583-bib-0011] Gelman, A. , Stern, H. S. , Carlin, J. B. , Dunson, D. B. , Vehtari, A. , & Rubin, D. B. (2013). Bayesian data analysis (3rd ed.). Boca Raton, FL: Chapman and Hall/CRC Press.

[ece36583-bib-0012] Gibson, D. , Blomberg, E. J. , & Sedinger, J. S. (2016). Evaluating vegetation effects on animal demographics: The role of plant phenology and sampling bias. Ecology and Evolution, 6, 3621–3631. 10.1002/ece3.2148 27148444 PMC4848082

[ece36583-bib-0013] Gill, F. B. (2007). Ornithology (3rd ed.). New York, NY: W.H. Freeman & Company.

[ece36583-bib-0014] Gosselink, T. E. , Van Deelen, T. R. , Warner, R. E. , & Joselyn, M. G. (2003). Temporal habitat partitioning and spatial use of coyotes and red foxes in east‐central Illinois. The Journal of Wildlife Management, 67, 90–103.

[ece36583-bib-0015] Healy, W. M. (1992). Population influences: Environment In Dickson J. G. (Ed.), The wild turkey: Biology and management (pp. 129–143). Harrisburg, PA: Stackpole Books.

[ece36583-bib-0016] Healy, W. M. , & Nenno, E. S. (1985). Effect of weather on wild turkey poult survival. Proceedings of the National Wild Turkey Symposium, 5, 91–101.

[ece36583-bib-0017] Hellerstein, D. M. (2017). The US Conservation Reserve Program: The evolution of an enrollment mechanism. Land Use Policy, 63, 601–610. 10.1016/j.landusepol.2015.07.017

[ece36583-bib-0018] Hinton, J. W. , van Manen, F. T. , & Chamberlain, M. J. (2015). Space use and habitat selection by resident and transient coyotes (Canis latrans). PLoS One, 10(7), e0132203. 26148130 10.1371/journal.pone.0132203 PMC4493083

[ece36583-bib-0019] Hubbard, M. W. , Garner, D. L. , & Klaas, E. E. (1999). Wild turkey poult survival in southcentral Iowa. The Journal of Wildlife Management, 63, 199–203. 10.2307/3802501

[ece36583-bib-0020] Huxoll, C. (2016). Annual report big game harvest projections. South Dakota game report No. 2017‐06. Pierre, SD: South Dakota Department of Game, Fish, and Parks.

[ece36583-bib-0021] Isabelle, J. L. , Conway, W. C. , Comer, C. E. , Calkins, G. E. , & Hardin, J. B. (2016). Reproductive ecology and nest‐site selection of eastern wild turkeys translocated to east Texas. Wildlife Society Bulletin, 40, 88–96. 10.1002/wsb.632

[ece36583-bib-0022] Johnson, J. R. , & Larson, G. E. (2007). Grassland plants of South Dakota and the northern great plains. Brookings, SD: South Dakota State University College of Agriculture & Biological Sciences and South Dakota Agricultural Experiment Station.

[ece36583-bib-0023] Johnson, R. R. , Higgins, K. F. , & Hubbard, D. E. (1995). Using soils to delineate South Dakota physiographic regions. Great Plains Research, 5, 309–322.

[ece36583-bib-0024] Kellner, K. (2018). jagsUI: A wrapper around RJAGS to streamline JAGS analyses. R package version 1.4.9. Retrieved from https://CRAN.R‐project.org/package=jagsUI

[ece36583-bib-0025] Knupp Moore, P. M. , & Flake, L. D. (1994). Forest characteristics in eastern and central South Dakota. Proceedings of the South Dakota Academy of Science, 73, 163–174.

[ece36583-bib-0026] Leatherberry, E. C. , Piva, R. J. , & Josten, G. J. (2000). South Dakota's forest resources outside the Black Hills National Forest, 1996. St. Paul, MN: North Central Research Station, USDA Forest Service.

[ece36583-bib-0027] Lehman, C. P. , Flake, L. D. , Leif, A. P. , & Shields, R. D. (2001). Comparative survival and reproduction of sympatric eastern and Rio Grande wild turkey females in northeastern South Dakota. Proceedings of the National Wild Turkey Symposium, 8, 123–125.

[ece36583-bib-0028] Lehman, C. P. , Flake, L. D. , Rumble, M. A. , & Thompson, D. J. (2008). Merriam's turkey poult survival in the Black Hills, South Dakota. Intermountain Journal of Sciences, 14, 78–88.

[ece36583-bib-0029] Lehman, C. P. , Rumble, M. A. , Flake, L. D. , & Thompson, D. J. (2008). Merriam's turkey nest survival and factors affecting nest predation by mammals. The Journal of Wildlife Management, 72, 1765–1774. 10.2193/2007-519

[ece36583-bib-0030] Lutz, R. S. , & Crawford, J. A. (1987). Reproductive success and nesting habitat of Merriam's wild turkeys in Oregon. The Journal of Wildlife Management, 51, 783–787. 10.2307/3801740

[ece36583-bib-0031] McConnell, M. D. , Monroe, A. P. , Burger, L. W. Jr , & Martin, J. A. (2017). Timing of nest vegetation measurement may obscure adaptive significance of nest‐site characteristics: A simulation study. Ecology and Evolution, 7, 1259–1270. 10.1002/ece3.2767 28303194 PMC5306001

[ece36583-bib-0032] McRoberts, J. T. , Wallace, M. C. , & Eaton, S. W. (2014). Wild Turkey (*Meleagris gallopavo*), version 2.0 In Poole A. F. (Ed.), The birds of North America. Ithaca, NY: Cornell Lab of Ornithology 10.2173/bna.22

[ece36583-bib-0033] Menne, M. J. , Durre, I. , Korzeniewski, B. , McNeal, S. , Thomas, K. , Yin, X. , … Houston, T. G. (2012). Global historical climatology network ‐ daily (GHCN‐Daily), Version 3.26. Asheville, NC: NOAA National Climatic Data Center 10.7289/V5D21VHZ

[ece36583-bib-0034] Miller, K. F. , Kempf, L. S. , & Koopman, V. F. (1979). Soil survey of grant county, South Dakota. Washington, DC: USDA Soil Conservation Service.

[ece36583-bib-0035] Mills, L. S. (2007). Conservation of wildlife populations: Demography, genetics, and management. Malden, MA: Blackwell Publishing.

[ece36583-bib-0036] Natural Resources Conservation Service [NRCS] (1999). Grassland birds. Fish and wildlife habitat management leaflet number 8. Madison, MS: Natural Resources Conservation Service.

[ece36583-bib-0037] Paisley, R. N. , Wright, R. G. , Kubisiak, J. F. , & Rolley, R. E. (1998). Reproductive ecology of eastern wild turkeys in southwestern Wisconsin. The Journal of Wildlife Management, 62, 911–916. 10.2307/3802542

[ece36583-bib-0038] Perlut, N. G. , Strong, A. M. , & Alexander, T. J. (2011). A model for integrating wildlife science and agri‐environmental policy in the conservation of declining species. Journal of Wildlife Management, 75, 1657–1663. 10.1002/jwmg.199

[ece36583-bib-0039] Plummer, M. (2003). JAGS: A program for analysis of Bayesian graphical models using Gibbs sampling. Proceedings of the 3rd international workshop on distributed statistical computing, Vienna, Austria.

[ece36583-bib-0040] Pollentier, C. D. , Hull, S. D. , & Lutz, R. S. (2014). Eastern wild turkey demography: sensitivity of vital rates between landscapes. The Journal of Wildlife Management, 78, 1372–1382. 10.1002/jwmg.787

[ece36583-bib-0041] Pollentier, C. D. , Lutz, R. S. , & Hull, S. D. (2014). Survival and productivity of eastern wild turkey females in contrasting landscapes in Wisconsin. The Journal of Wildlife Management, 78, 985–996. 10.1002/jwmg.749

[ece36583-bib-0042] Porter, W. F. , Nelson, G. C. , & Mattson, K. (1983). Effects of winter conditions on reproduction in a northern wild turkey population. The Journal of Wildlife Management, 47, 281–290. 10.2307/3808500

[ece36583-bib-0043] R Core Team (2018). R: A Language and Environment for Statistical Computing. Vienna, Austria: R Foundation for Statistical Computing Retrieved from https://www.R‐project.org/

[ece36583-bib-0044] Reitsma, K. D. , Clay, D. E. , Carlson, C. G. , Dunn, B. H. , Smart, A. J. , Wright, D. L. , & Clay, S. A. (2014). Estimated South Dakota land use change from 2006 to 2012. Agronomy, Horticulture and Plant Science Faculty Publications, 18 Retrieved from https://openprairie.sdstate.edu/plant_faculty_pubs/18

[ece36583-bib-0045] Robel, R. J. , Briggs, J. N. , Dayton, A. D. , & Hulbert, L. C. (1970). Relationships between visual obstruction measurements and weight of grassland vegetation. Journal of Range Management, 23, 295–297. 10.2307/3896225

[ece36583-bib-0046] Roberts, S. D. , Coffey, J. M. , & Porter, W. F. (1995). Survival and reproduction of female wild turkeys in New York. The Journal of Wildlife Management, 59, 437–447. 10.2307/3802449

[ece36583-bib-0047] Roberts, S. D. , & Porter, W. F. (1998a). Influence of temperature and precipitation on survival of wild turkey poults. The Journal of Wildlife Management, 62, 1499–1505. 10.2307/3802016

[ece36583-bib-0048] Roberts, S. D. , & Porter, W. F. (1998b). Relation between weather and survival of wild turkey nests. The Journal of Wildlife Management, 62, 1492–1498. 10.2307/3802015

[ece36583-bib-0049] Rolley, R. E. , Kubisiak, J. F. , Paisley, R. N. , & Wright, R. G. (1998). Wild turkey population dynamics in southwestern Wisconsin. The Journal of Wildlife Management, 62, 917–924. 10.2307/3802543

[ece36583-bib-0050] Royle, J. A. , & Dorazio, R. M. (2008). Hierarchical modeling and inference in ecology: The analysis of data from populations, metapopulations, and communities. San Diego, CA: Academic Press.

[ece36583-bib-0051] Sæther, B.‐E. , & Bakke, Ø. (2000). Avian life history variation and contribution of demographic traits to the population growth rate. Ecology, 81, 642–653. 10.1890/0012-9658(2000)081[0642:ALHVAC]2.0.CO;2

[ece36583-bib-0052] Schmidt‐Nielsen, K. (1997). Animal physiology: Adaptation and environment. Cambridge, UK: Cambridge University Press.

[ece36583-bib-0053] Shields, R. D. , & Flake, L. D. (2006). Survival and reproduction of translocated eastern wild turkeys in a sparsely wooded landscape in north eastern South Dakota. Western North American Naturalist, 66, 298–309. 10.3398/1527-0904(2006)66[298:SAROTE]2.0.CO;2

[ece36583-bib-0054] Smith, J. T. , Tack, J. D. , Doherty, K. E. , Allred, B. W. , Maestas, J. D. , Berkeley, L. I. , … Naugle, D. E. (2018). Phenology largely explains taller grass at successful nests in greater sage‐grouse. Ecology and Evolution, 8, 356–364. 10.1002/ece3.3679 29321877 PMC5756841

[ece36583-bib-0055] South Dakota Department of Transportation [SDDOT] (2017). Downloadable local roads. Pierre, SD: South Dakota Department of Transportation Retrieved from https://opendata2017‐09‐18t192802468z‐sdbit.opendata.arcgis.com/datasets/f63b4835c188471686326fb9f2b64359_0

[ece36583-bib-0056] Spears, B. L. , Wallace, M. C. , Ballard, W. B. , Phillips, R. S. , Holdstock, D. P. , Brunjes, J. H. , … Gipson, P. S. (2007). Habitat use and survival of preflight wild turkey broods. The Journal of Wildlife Management, 71, 69–81. 10.2193/2005-676

[ece36583-bib-0057] Switzer, C. T. , & Tucker, S. A. (2009). Survival, reproduction, home range, and habitat use of translocated eastern wild turkeys in the Wessington Hills, South Dakota. Game Report 2009‐07. Pierre, SD: South Dakota Department of Game, Fish and Parks.

[ece36583-bib-0058] Tigas, L. A. , Van Vuren, D. H. , & Sauvajot, R. M. (2002). Behavioral responses of bobcats and coyotes to habitat fragmentation and corridors in an urban environment. Biological Conservation, 108(3), 299–306.

[ece36583-bib-0059] Thompson, M. C. , & Delong, R. L. (1967). The use of cannon and rocket‐projected nets for trapping shorebirds. Bird‐Banding, 38, 214–218. 10.2307/4511387

[ece36583-bib-0060] USDA (2016). Change in CRP enrollment 2007–2016. Washington, DC: USDA Retrieved from https://www.fsa.usda.gov/Assets/USDA‐FSA‐Public/usdafiles/Conservation/PDF/ChangeInCRPAcreagefrom2007_2016.pdf

[ece36583-bib-0061] Vander Haegen, W. M. , Dodge, W. E. , & Sayre, M. W. (1988). Factors affecting productivity in a northern wild turkey population. The Journal of Wildlife Management, 51, 127–133. 10.2307/3801072

[ece36583-bib-0062] Vangilder, L. D. , & Kurzejeski, E. W. (1995). Population ecology of the eastern wild turkey in northern Missouri. Wildlife Monographs, 130, 1–50.

[ece36583-bib-0063] Vangilder, L. D. , Kurzejeski, E. W. , Kimmel‐Truitt, V. L. , & Lewis, J. B. (1987). Reproductive parameters of wild turkey hens in north Missouri. The Journal of Wildlife Management, 51, 535–540. 10.2307/3801265

[ece36583-bib-0064] Williams, L. E. Jr (1961). Notes on wing molt in the yearling wild turkey. The Journal of Wildlife Management, 25, 439–440. 10.2307/3798838

[ece36583-bib-0065] Wood, J. D. , Cohen, B. S. , Conner, L. M. , Collier, B. A. , & Chamberlain, M. J. (2019). Nest and brood site selection of eastern wild turkeys. Journal of Wildlife Management, 83, 192–204. 10.1002/jwmg.21562

[ece36583-bib-0066] Wright, R. G. , Paisley, R. N. , & Kubisiak, J. F. (1996). Survival of wild turkey hens in southwestern Wisconsin. Journal of Wildlife Management, 60, 313–320. 10.2307/3802230

[ece36583-bib-0067] Wunz, G. A. (1984). Rocket‐net innovations for capturing wild turkeys and waterfowl. Transactions of the Northeast Section of the Wildlife Society, 41, 219.

[ece36583-bib-0068] Yang, L. , Jin, S. , Danielson, P. , Homer, C. , Gass, L. , Bender, S. M. , … Funk, M. (2018). A new generation of the United States National Land Cover Database: Requirements, research priorities, design, and implementation strategies. ISPRS Journal of Photogrammetry and Remote Sensing, 146, 108–123.

[ece36583-bib-0069] Yarnall, M. J. , Litt, A. R. , & Lehman, C. P. (2019). Timing of vegetation sampling does not influence associations between visual obstruction and turkey nest survival in a montane forest. Ecology and Evolution, 9, 11791–11798. 10.1002/ece3.5681 31695888 PMC6822050

